# Supplementary material for: mPFC spindle cycles organize sparse thalamic activation and recently active CA1 cells during non-REM sleep
Source: eLife. 2020 Jun 11;9:e48881. doi: 10.7554/eLife.48881 (PMC7319772; doi:10.7554/eLife.48881)
Supplement: Transparent reporting form [file elife-48881-transrepform.docx]

***eLife’s* transparent reporting form**

We encourage authors to provide detailed information *within their submission* to facilitate the interpretation and replication of experiments. Authors can upload supporting documentation to indicate the use of appropriate reporting guidelines for health-related research (see [EQUATOR Network](http://www.equator-network.org/%20)), life science research (see the [BioSharing Information Resource](https://biosharing.org/)), or the [ARRIVE guidelines](http://www.plosbiology.org/article/info:doi/10.1371/journal.pbio.1000412) for reporting work involving animal research. Where applicable, authors should refer to any relevant reporting standards documents in this form.

If you have any questions, please consult our Journal Policies and/or contact us: [editorial@elifesciences.org](mailto:editorial@elifesciences.org).

**Sample-size estimation**

- You should state whether an appropriate sample size was computed when the study was being designed
- You should state the statistical method of sample size computation and any required assumptions
- If no explicit power analysis was used, you should describe how you decided what sample (replicate) size (number) to use

Please outline where this information can be found within the submission (e.g., sections or figure legends), or explain why this information doesn’t apply to your submission:

Sample sizes are provided through the text to document each analyses. Because the goal of these experiments was to characterize baseline interactions between brain regions, there were no experimental groups in the design and no power analysis calculation.

**Replicates**

- You should report how often each experiment was performed
- You should include a definition of biological versus technical replication
- The data obtained should be provided and sufficient information should be provided to indicate the number of independent biological and/or technical replicates
- If you encountered any outliers, you should describe how these were handled
- Criteria for exclusion/inclusion of data should be clearly stated
- High-throughput sequence data should be uploaded before submission, with a private link for reviewers provided (these are available from both GEO and ArrayExpress)

Please outline where this information can be found within the submission (e.g., sections or figure legends), or explain why this information doesn’t apply to your submission:

Criteria for exclusion/inclusion are included in the methods section under the ‘Selection of Electrodes and Sessions for Analyses’ sub-section, which also explains procedures regarding biological vs technical replication. One of the major inclusion criteria in electrophysiology experiments is the need for unambiguous identification of electrode location; the raw histology pictures with this information are provided in the Figure 1- figure Supplement 1. Something that is as important (but documented less often), is the estimated trajectory travelled by the electrodes through brain tissue from the start to the end of recording (also provide in the Supplementary figures).

**Statistical reporting**

- Statistical analysis methods should be described and justified
- Raw data should be presented in figures whenever informative to do so (typically when N per group is less than 10)
- For each experiment, you should identify the statistical tests used, exact values of N, definitions of center, methods of multiple test correction, and dispersion and precision measures (e.g., mean, median, SD, SEM, confidence intervals; and, for the major substantive results, a measure of effect size (e.g., Pearson's r, Cohen's d)
- Report exact p-values wherever possible alongside the summary statistics and 95% confidence intervals. These should be reported for all key questions and not only when the p-value is less than 0.05.

Please outline where this information can be found within the submission (e.g., sections or figure legends), or explain why this information doesn’t apply to your submission:

Statistical analysis methods are reported in the Methods section under ‘Correlation quantifications’ and ‘Phase-locking analyses’, and on a case-by-case basis through the text. Figure 1- figure Supplement 3 illustrates with an example the process to estimate the distribution under the Null hypothesis used to evaluate the significance of baseline correlations. P-values are reported through the manuscript and the full population distribution of statistics is shown using whisker plots on all population figures.

(For large datasets, or papers with a very large number of statistical tests, you may upload a single table file with tests, Ns, etc., with reference to sections in the manuscript.)

**Group allocation**

- Indicate how samples were allocated into experimental groups (in the case of clinical studies, please specify allocation to treatment method); if randomization was used, please also state if restricted randomization was applied
- Indicate if masking was used during group allocation, data collection and/or data analysis

Please outline where this information can be found within the submission (e.g., sections or figure legends), or explain why this information doesn’t apply to your submission:

There were no animal groups in our study. Grouping of cells or sleep events for analysis of functionally classified data is reported in each section.

**Additional data files (“source data”)**

- We encourage you to upload relevant additional data files, such as numerical data that are represented as a graph in a figure, or as a summary table
- Where provided, these should be in the most useful format, and they can be uploaded as “Source data” files linked to a main figure or table
- Include model definition files including the full list of parameters used
- Include code used for data analysis (e.g., R, MatLab)
- Avoid stating that data files are “available upon request”

Please indicate the figures or tables for which source data files have been provided:

Data files and code are available through the CRCNS website (data set ‘HC-24’); this includes data sets with raw data (LFP and units) recorded from 3 sessions from 3 rats, derived data (such as sleep-related events, like K-complexes, ripples and spindle cycles) as well as matlab code used to obtain the main findings in the manuscript.
